# Cumulative life course impairment in patients with dermatological diseases, with a focus on psoriasis^⋆^

**DOI:** 10.1016/j.abd.2023.08.006

**Published:** 2023-12-21

**Authors:** Ricardo Romiti, Renata Ferreira Magalhães, Gleison Vieira Duarte

**Affiliations:** a Department of Dermatology, Hospital das Clínicas, Universidade de São Paulo, SP, Brazil; b Discipline of Dermatology, Faculty of Medical Sciences, Universidade Estadual de Campinas, SP, Brazil; c Instituto Bahiano de Imunoterapias, Salvador, BA, Brazil

**Keywords:** Life, Psoriasis, Skin diseases

## Abstract

The concept of “Cumulative Life Course Impairment” (CLCI) characterizes the set of factors harmful to the lives of patients resulting from the stigma and physical and psychological impairment associated with different chronic diseases, which can accumulate irreversibly over the course of patients lives. The sum of these factors often makes it impossible for these individuals to enjoy their lives fully, intensely and adequately. On the other hand, CLCI also incorporates coping strategies, including external factors and personality characteristics, which may act as modulating or protective factors of vulnerability to the CLCI. Although psoriasis is the most studied dermatological disease in relation to its impact on quality of life and CLCI, several other chronic inflammatory diseases such as atopic dermatitis, hidradenitis suppurativa, and alopecia areata have also been evaluated in relation to the magnitude of the damage to patients lives.

## Introduction

Chronic and recurrent dermatoses have an impact on the physical, psychological and social well-being of patients, and can cause irreversible damage throughout their lives. Based on this observation, the concept of Cumulative Life Course Impairment (CLCI) was established, aiming to evaluate the main impairments accumulated during patients lives, understand the progressive impact of certain diseases, identify the individuals most likely to be affected, and thus establish appropriate coping strategies for each patient.

Psoriasis is the most studied dermatological disease in relation to its impact on quality of life and CLCI. On the other hand, several other chronic inflammatory diseases such as atopic dermatitis, hidradenitis suppurativa, and alopecia areata have also been evaluated in relation to the magnitude of damage to patients lives.^1^, ^2^

In turn, recent projects aim to establish appropriate assessment tools to measure CLCI, identify individuals most likely to develop irreversible impairments associated with different chronic diseases and establish the best coping strategies.^3^, ^4^

This article aims to objectively define what the cumulative life course impairment is, who is at greatest risk of being affected by it, and what the role of the dermatologist is in multidisciplinary care for patients with chronic dermatological diseases.

## Historical aspects

The concept of CLCI is rooted in the areas of psychology and sociology based on the analysis of factors that negatively influence the lives of patients with chronic diseases and how protective factors and risk factors can interact.^4^

The most striking difference between CLCI and Health-Related Quality of Life (HRQoL) studies – is the fact that the latter assess the impact of the disease on quality of life in a cross-sectional way or only over a short period of time, while CLCI aims to longitudinally analyze the impact throughout patients lives.

Kimball et al. (2010) originally applied the concept of CLCI to patients with psoriasis, counterbalancing not only the stigma associated with the disease, which ends up impacting self-perception, self-confidence and individual behavior but also physical comorbidities (psoriatic arthritis, metabolic syndrome, and cardiovascular disease, etc.), psychological comorbidities (anxiety, depression and suicidal ideation) with strategies that may have a modulating effect on such impairment, such as appropriate therapeutic conducts, social support from relatives, friends and co-workers and support groups for patients.^5^

The authors will discuss the main factors involved in the concept of CLCI, their possible interactions and the dermatologist role in the multidisciplinary care offered to patients with chronic dermatological diseases.

## Risk factors for CLCI in patients with psoriasis

The identification of risk factors for CLCI is based on finding one or several reliable valid parameters, which can predict the long-term outcomes of the disease over the course of the individual life. However, there is yet no validated instrument for assessing CLCI.^6^

The impact of psoriasis on CLCI depends on the combination of several factors, with the main ones being classified as follows:

Factors related to psoriasis: early age of onset; physical factors: pruritus, pain, arthritis (disease severity); lack of treatment or difficulties with adequate treatment.

Factors related to comorbidities: psychological factors: depression, anxiety, substance abuse; physical factors: obesity, cardiovascular, intestinal and other diseases.

Social factors: stigmatization; personality, coping mechanisms, social support, and treatment; low level of schooling, fewer training and work opportunities; lower financial gain.

The cumulative effect of exposures (psoriasis, stigmatization, physical and psychological comorbidities) that occur at specific moments throughout patients lives add up to the point of harming their potential to have a full and happy life.^5^

A systematic review of the literature to map the risk factors for CLCI in psoriasis showed that 13 risk factors were explored.^1^

Greater disease severity is a risk factor for worse overall health, with the development of depression and other psychiatric disorders and more comorbidities, such as cardiovascular disorders and malignancies. In turn, cardiovascular disorders and diabetes are risk factors for other problems, such as sexual dysfunction. The female gender, younger and older patients, early onset of the disease, low level of schooling, smoking, and systemic treatment, as well as a high number of hospitalizations and worse overall health status, are factors that generate a large cumulative psychological burden over time.^1^, ^7^, ^8^

## Major Life Changes Decisions ‒ MLCDs

Chronic diseases have a major impact on critical decisions in patients lives. A study of 50 patients with chronic dermatological diseases, including 16 patients with psoriasis, showed that the dermatosis influenced career choice in 66%, relationships in 52%, education in 44%, moving abroad in 32%, having children in 22%, housing in 14% and moving to another city in 12%.^9^, ^10^, ^11^

CLCI is directly influenced by the timing of psoriasis onset, according to the life course epidemiology theory, which postulates that the effect exerted by exposures on health outcomes depends on the timing of exposure. In some cases, an exposure may only have an effect if it occurs during a critical period of development and this effect may be irreversible.^12^

The concepts of sensitive and critical periods also apply. The early onset of psoriasis during adolescence (critical period) and early adulthood (sensitive period), when patients are consolidating their personality, establishing social contacts, and deciding on higher education and career planning, will have a greater impact throughout life.^13^

Likewise, exposure to comorbidities such as psoriatic arthritis during the sensitive period of early adulthood may result in lower earnings (e.g., need to change careers or retire early), leading to other exposures such as anxiety, compromised social life, depression, symptom worsening and low adherence to medical follow-up and treatment, all of which interact cumulatively.^14^

Psoriatic arthritis is a common condition among patients with psoriasis, with an estimated prevalence between 24% and 33%, which can further physically weaken individuals. The impairment of these patients quality of life is much greater when compared to patients with psoriasis without joint disease.^15^, ^16^, ^17^

Stigmatization, according to the dictionary, means the action or effect of stigmatizing, of branding with stigma, with a hot iron. The term is used to define the act of negatively branding something or someone.^18^ It involves the attribution of discrediting biological or social perceptions to a person, differentiating a person from others in a society. The feeling of stigmatization is common in dermatological patients, with diseases such as psoriasis, vitiligo and leprosy.^19^

A survey of 8,338 psoriasis patients from 31 countries showed that 84% experienced discrimination related to psoriasis, with had a negative impact on their work and overall health. Patients with psoriasis may experience social and psychological difficulties in their daily lives, especially when they have to expose their bodies.^20^

The social stigmatization faced by patients with psoriasis is greater than what is seen in other skin diseases, causing serious lifelong emotional disorders. The presence of lesions in visible areas can lead to open rejection by other people, which inhibits attending public environments and having social interactions.^19^

Magnetic resonance imaging studies show that the brains of patients with psoriasis have attenuated responses to facial images of disgust. Patients become used to rejection and are more sensitive to the negative attitudes of those around them.^21^

The self-image and self-confidence of patients with psoriasis become increasingly damaged over time. Relationships can be negatively affected and studies show that the quality of life of family members of the patient with psoriasis is also compromised.^22^

Patients tend to have anxiety related to sexual relations, regardless of disease severity. Almost half of the patients with psoriasis feel unattractive, have difficulty getting married, and have higher divorce rates.^23^

The decision to marry and have children is another factor that may contribute to a fuller life and can influence the CLCI, especially for young women. There are concerns such as the risk of having descendants with the disease, fear of worsening the disease during pregnancy, of having complications for the fetus due to the treatment, having to stop treatment due to the risk of teratogenicity.^12^

Stigmatization, reduced self-confidence, avoidance of public places, and relationship dysfunction combine to create a hostile social environment causing psychological comorbidities. The data show anxiety and depression in a large percentage of patients, regardless of disease severity. The high rate of suicidal ideation in up to 10% of patients with psoriasis is a matter of concern. Mental anguish often does not decrease with the disappearance of skin lesions, which shows that adequate treatment may not be sufficient to control this factor.^24^, ^25^, ^26^, ^27^, ^28^, ^29^ Different studies in Brazilian populations show 19% prevalence rates of depression and 36% of anxiety, and up to 69.8% of both in patients with psoriatic arthritis.^30^, ^31^, ^32^

A study of 1,125 patients showed that the severity of psoriasis was significantly associated with the presence of physical inactivity and pain, anxiety, and depression.^33^

The association between psoriasis and depressive mood disorders may be responsible for the high frequency of maladaptive behaviors, such as social phobia, smoking, and alcoholism. The severity of psoriasis and psychosocial rejection, anxiety, and depression have been associated with increased alcohol use by patients. Patients with psoriasis are more likely to continue smoking as they age. Smoking and alcoholism aggravate the comorbidities and increase morbidity and mortality from the disease, reduce adherence to treatment, and feed a vicious cycle. Lifestyle changes, restricted diets, among others, can also be another stressful factor for the patient.^34^

There are negative effects on the economic life of patients with psoriasis. Increased severity of psoriasis is related to lower income and less likelihood of being employed full-time. Access to healthcare can also be negatively affected by the disease and the ability to perform well at work. Finlay and Coles demonstrated that one-third of unemployed patients attribute not working to psoriasis.^35^

Those who are employed report more lost workdays and decreased productivity compared to those without psoriasis. More than 20% of the patients report that psoriasis affected their career choice.^34^, ^36^, ^37^, ^38^, ^39^

In a Brazilian study, the estimated annual cost per patient with psoriasis was US$4,034. Direct medical costs represented 87.7% of this estimate.^32^

A compilation of the different factors that influence cumulative life course impairment and their respective implications are listed in Table 1.^40^

**Table 1:** Factors that influence Cumulative Life Course Impairment.

Factor		Implication
Underlying disease	Disease severity	Psoriasis lesions cause physical discomfort because they crack the skin, cause pruritus and bleeding, desquamate, stain, and cause social embarrassment.
		Lower overall health status, depression and other psychological changes, cardiovascular disease, and malignancies are related to more severe illness.
	Special characteristics of psoriasis	Nail disease or disease located in visible areas and psoriatic arthritis are associated with more difficulty in dealing with the disease.
	Gender and age	Younger and older people are the most impacted.
		Women have concerns about pregnancy, its risks, fear of stopping treatment or worsening the disease or bringing complications to the fetus.
	Early symptoms	In childhood or adolescence, the most vulnerable phase, there are fewer adaptive mechanisms to protect against personality damage. Living with negative reactions from classmates influences academic performance, training, opportunities and career choices, first social contacts and partnerships.
		It also means a longer duration of the disease and more time to experience its complications.
	Chronicity	The perception of prolonged illness requires adaptation to living with psoriasis and can have a major negative impact.
	Disease duration	It may indicate a longer period of exposure to factors that are potentially harmful throughout the course of life. Related to the concept of chronicity.
	Treatment	The obligation to maintain medication for the long term can cause anxiety, discomfort, lack of stimulation and reduce adherence. The cost and fear of adverse events also have a negative influence.
Comorbidities	Obesity	It contributes to low self-esteem. Individuals stop performing physical activities, due to pain or embarrassment, or depression, which contributes to weight gain. The systemic inflammation of psoriasis itself is associated with a greater chance of obesity and metabolic syndrome.
	Cardiovascular comorbidities	They affect several functions, including performing exercise, physical limitations, which may be aggravated by pain, indirectly compromising sexual function.
	Other associated diseases	Inflammatory bowel disease and arthritis are factors that add up, aggravating low self-esteem, the need to seek health services, undergoing exams and using medications, causing more limitations.
	Higher number of hospitalizations	Patients may have severe forms such as pustular or erythrodermic psoriasis, complications from comorbidities and treatments, requiring hospitalization.
Psychosocial	Low level of schooling	The individual has difficulty in school relationships, with classmates and teachers, difficulty in interacting, concentrating, missing school to attend appointments or undergo exams, social embarrassment due to the presence of lesions. There is a tendency to abandon studies or not invest in labor training, especially in the lower social classes.
	Stigmatization	Feeling of being stigmatized and disregarded by others.
	Lack of social support	Participation of family, friends or other caregivers, with psychological and material support. Improves coping with chronic illnesses in general.
	Negative impact on professional life	Lack of schooling and adequate training, greater number of days lost at work, low productivity due to pain or skin lesions.
	Negative mood	Anger, depression, sadness, helplessness and loss of autonomy.
	Coping strategies	Well-described correlation between psychological burden and coping behavior in psoriasis. Negative coping is related to anxiety, depression and anguish due to the disease.
	Quality of life	Influenced by important predictors: time needed for treatment, social anxiety, negative coping, clinical severity, satisfaction with treatment.
	Risk behaviors	Smoking, alcoholism, drug addiction.
		Compulsive food addiction.
		They are associated with more anxiety and lack of a coping strategy. They worsen comorbidities such as obesity and increase cardiovascular risk.

Adapted source: Warren RB et al., 2011.^40^

## Ways of handling the disease: coping strategies

CLCI postulates that the way one faces disease, that is, coping strategies, can have a moderating effect on the cumulative impact of psoriasis. Adaptive coping strategies (e.g., social support, information seeking, and adherence to treatment regimens) can reduce the burden of the disease. Negative coping strategies (e.g., behavioral disengagement, expressing emotions, denial, and alcohol or substance use) can impact the quality of life, morbidity, and mortality associated with the disease.

The way one views the disease interacts with the biopsychosocial spheres over time and can have a positive or negative effect on the cumulative impact of psoriasis, for instance, leading to denial or victimization in relation to the disease.^5^, ^40^, ^41^, ^42^

**Figure 1:**
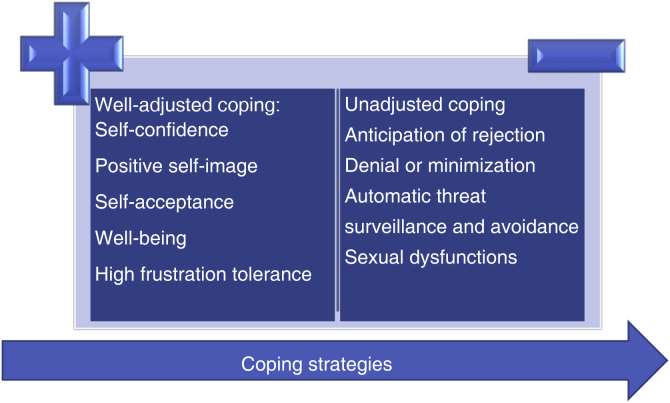
Benefits of positive, well-adjusted coping versus counterproductive negative and passive coping strategies in the long term.

The benefits of positive and well-adjusted coping when compared to counterproductive negative and passive coping strategies in the long term are outlined in Fig. 1.^4^, ^25^

## How to address in practice the risk of cumulative and irreversible damage to patients lives – the dermatologist role

Initially, the most vulnerable groups and the main risk factors for the occurrence of damage must be identified.^5^

As CLCI is more comprehensive than the classic dimensions of quality of life (physical, social and psychological), its assessment must be fundamentally individualized, and consequently, treatment will be customized.^40^ The impact of health on quality of life may be reduced over time, suggesting that patients may adjust their internal standards and values to accommodate the realities of their condition, as opposed to the cumulative impact on life, which encompasses economic, social, employability and patient family status.^14^, ^40^

Appropriate and early treatment of the disease can have a preventive role and allow the individual to reach full life potential.^4^ In these patients, advice on the need for social support, safe sources of information and adherence to treatment regimens is crucial, and can even prevent inappropriate coping strategies, such as alcohol consumption and cigarette smoking.^1^, ^34^

Understanding the patient perspective on the disease, clarifying the benefits and limitations of alternative diets and treatments can also prevent unproven or ineffective strategies. It is known that up to 51% of the patients with psoriasis report using complementary and alternative medicine (CAM) in their treatment regimen, but studies suggest that up to 42.3% did not disclose this to their doctor.^43^ In the Brazilian scenario, it is known that there is an invisibility of CAM, which is harmful not only to scientific knowledge but also to patients, who often do not disclose the other forms of care they use, taking risks with the aim of not exposing themselves to further social humiliation.^44^

Moreover, a survey identified that only 26% of the patients with severe psoriasis were treated with systemic therapy, phototherapy, or both, while 35% received topical therapy and 39% were untreated, which shows that psoriasis remains an undertreated disease.^45^ It is also known that despite being known for decades, the comorbidities of psoriasis tend to be underdiagnosed and undertreated.^46^

Considering both components that together lead to CLCI, the authors divided possible strategies for each of them.

### Role of the dermatologist in improving coping strategies and external factors

1. Validate (identify) the patient subjective perspective^47^

1.1. Identify the present impact on quality of life through the DLQI.

1.2. Greater expansion of QoL scales in children and adolescents – DLQI child – whose verbalization of emotions is more difficult.^48^

1.3. Use the cutaneous body image score to psychometrically assess individual satisfaction with appearance.^49^

2. Inquire about stigma^50^

2.1. Surveillance of bullying and cyberbullying in children and adolescents.

2.2. Advice on how to redirect attention to child bullying or cyberbullying victims.

2.3. Connect families with support groups.

2.4. Referral for professional support when necessary.

3. Identify potential past impacts on employment and income^1^, ^40^

3.1. Presenteeism, absenteeism and loss of productivity.

3.2. Unemployment, early retirement, sick pay and advancement/opportunities at work.

4. Identification of maladapted support systems^4^

4.1. Denial, fatalism, minimization, feeling of helplessness and reduction in activities.

4.2. Prolonged social avoidance leading to disconnection.

5. Identify potential impacts or risks to family organization^40^

5.1. Divorce.

5.2. Desire to conceive children.

5.3. Delay of conception in women of childbearing age due to the disease and use of appropriate systemic therapies in women of childbearing age at risk of an unplanned pregnancy.^51^

5.4. Couples sexual life.

These multiple dimensions of life can be irreversibly affected by psoriasis and other chronic diseases, but early detection of maladjusted coping systems can prevent future damage. For example, Rational Emotive-Behavioral Therapy (REBT) theorizes that people, to minimize the damage caused by an event, need to work on unconditional self-acceptance, unconditional hetero-acceptance, unconditional acceptance of life, and a philosophy of high tolerance to frustration. Considering the need for multidisciplinary support for patients with chronic diseases, strategies for repositioning the interpretations one makes in the face of life events can be important in situations such as bullying, or in the formation of a positive body image in the presence of skin and nail lesions, etc., very much in line with the famous quote from Epictetus – “We are disturbed not by events, but by the views which we have of them”.^52^

### Role of the dermatologist in the biomedical aspect

1. Treatment of psoriasis according to local goals

1.1. For psoriasis vulgaris, reach a minimum PASI of 75 in patients on systemic therapies, ideally PASI 90 or absolute PASI < 3, according to the psoriasis consensus of the Brazilian Society of Dermatology (SBD, *Sociedade Brasileira de Dermatologia*), in patients using immunobiologicals.^47^

1.2. Surveillance and assertive treatment of special areas, are associated with a greater chance of impact on quality of life.^47^

1.3. Early diagnosis of pustular psoriasis, intensive monitoring and rapid treatment are strategies to minimize the risk of hospitalization and death.^53^

2. Global assessment of mental health and detection of comorbidities – anxiety, depression, suicidal ideation.^29^

3. Early detection of psoriatic arthritis – use of screening questionnaires or routine periodic assessment of articular signs and symptoms.^54^

4. Detection of other comorbidities that may influence the choice or anticipation of the use of immunobiologicals: hidradenitis suppurativa, multiple sclerosis, heart failure, inflammatory bowel disease, uveitis, obesity, neoplasms, infections and lupus erythematous.^1^

5. Detection of comorbidities associated with cardiovascular risk or potential years of life lost – metabolic syndrome, obesity, diabetes, cardiovascular or cerebrovascular disease, erectile dysfunction.^55^

6. Identification of risk factors associated with lifestyle and inadequate coping strategies – smoking and alcohol consumption – and more rigorous screening for depression and anxiety in patients with alcohol consumption problems.^5^

7. Monitoring of comorbidities associated with the therapeutic toxicity of psoriasis.^29^

**Figure 2:**
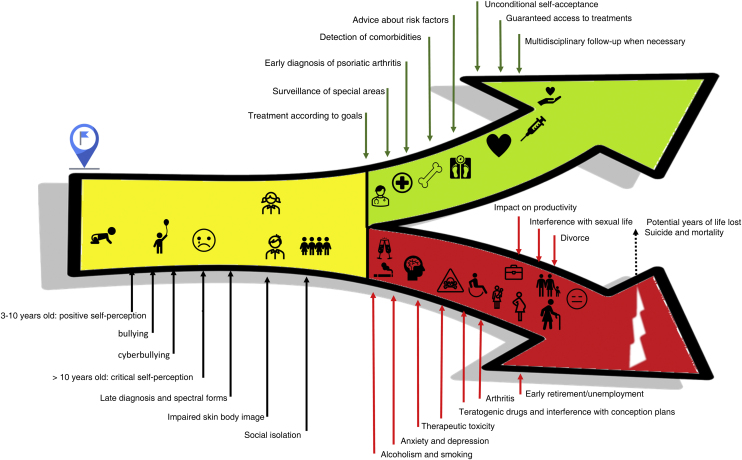
Multiple opportunities for intervention in different domains that promote high Cumulative Life Course Impairment (red) and the dermatologist role (green) in reducing potential irreversible damage in a critical period of development (childhood and adolescence, yellow) throughout life.

These strategies can be used in the different situations listed in Fig. 2, frequently experienced by patients with psoriasis, notably in its more severe forms, with a multisystemic characteristic and greater potential for classic and emerging comorbidities, related to treatment and lifestyle.^29^

## Final considerations

Aiming to minimize and fight the harmful factors resulting from the stigma and physical and psychological impairment inherent to different chronic dermatological diseases, which can accumulate irreversibly over the course of patients lives, different strategies must always be considered. The need for an individualized and multidisciplinary assessment, the choice of appropriate and early treatments as well as advice on the need for social support, safe sources of information and adherence to treatment regimens are crucial to achieve a full, intense and adequate experience. The authors highlight the importance that University Hospitals, without exception, offer specific outpatient clinics for the care and monitoring of these patients, as well as the relevance of belonging to a patient group, allowing mutual support and the exchange of experiences, all in line with the raising of awareness and attenuation the impact of CLCI.

## Financial support

None declared.

## Authors' contributions

Ricardo Romiti: Design and planning of the study; data collection, or analysis and interpretation of data; statistical analysis; drafting and editing of the manuscript or critical review of important intellectual content; collection, analysis, and interpretation of data; effective participation in research orientation; intellectual participation in the propaedeutic and/or therapeutic conduct of the studied cases; critical review of the literature; approval of the final version of the manuscript.

Renata Ferreira Magalhães: Design and planning of the study; data collection, or analysis and interpretation of data; statistical analysis; drafting and editing of the manuscript or critical review of important intellectual content; collection, analysis, and interpretation of data; effective participation in research orientation; intellectual participation in the propaedeutic and/or therapeutic conduct of the studied cases; critical review of the literature; approval of the final version of the manuscript.

Gleison Vieira Duarte: Design and planning of the study; data collection, or analysis and interpretation of data; statistical analysis; drafting and editing of the manuscript or critical review of important intellectual content; collection, analysis, and interpretation of data; effective participation in research orientation; intellectual participation in the propaedeutic and/or therapeutic conduct of the studied cases; critical review of the literature; approval of the final version of the manuscript.

## Conflicts of interest

Ricardo Romitti: Is/has worked as a scientific consultant, speaker, or clinical study investigator for Abbvie, Boehringer-Ingelheim, Bristol Myers Squibb. Eli-Lilly, Janssen, Leo‐Pharma, Galderma, Sanofi, Novartis, Pfizer, Sanofi, UCB.

Renata Ferreira Magalhães: Is/has worked as a scientific consultant, speaker, or clinical study investigator for Abbvie, Eli-Lilly, Janssen, Leo‐Pharma, Galderma, Sanofi, Novartis, Pfizer.

Gleison Vieira Duarte: Is/has worked as a scientific consultant, speaker, or clinical study investigator for Abbvie, Amgen, Bayer, Boehringer-Ingelheim, Bristol Myers Squibb, Eli-Lilly, Janssen, Leo‐Pharma, Galderma, Novartis, Pfizer, Sanofi, UCB.
